# Comparison of single or two dose veterinarian-administered protocols and daily at-home prescription canine otitis externa treatments

**DOI:** 10.18849/ve.v11i1.734

**Published:** 2026-02-18

**Authors:** Ashlee Minne+, Isabella Alarcon+, Garen Burch+, Stephanie Kay+, Osdel Macides+, Caroline Thompson+, Mariah K. Zeigler+

**Keywords:** AT HOME, CANINE, DOGS, EAR, OTITIS EXTERNA, OSURNIA®

## Abstract

**PICO question:**

In adult dogs, are single or two dose veterinarian-administered ear treatments as effective in the treatment of otitis externa as daily prescription at-home cleaning/medicating treatments?

**Clinical bottom line:**

**Category of research:**

Treatment.

**Number and type of study designs reviewed:**

Three randomised-controlled studies were critically appraised.

**Strength of evidence:**

Strong.

**Outcomes reported:**

All three studies demonstrated that both daily at-home cleaner/prescription medication and either single or two-dose veterinarian-administered ear treatments for canine otitis externa were effective. When comparing the final outcomes, there were no significant differences in the efficacy of either treatment option. One of the three studies reviewed did not note initial variations in pruritus levels within the first seven days, but by the end of each trial, both treatment protocols proved effective in reducing pruritus, pain, and cytology counts–including bacteria and fungi–with low relapse rates reported.

**Conclusion:**

Two dose veterinarian-administered ear treatments like Osurnia^®^ show comparable efficacy in reducing the treatment of otitis externa in adult dogs when compared to daily at home treatments. Both types of treatments demonstrated similar success rates, with no significant difference in recurrence rates across the studies.

How to apply this evidence in practice

The application of evidence into practice should take into account multiple factors, not limited to: individual clinical expertise, patient’s circumstances and owners’ values, country, location or clinic where you work, the individual case in front of you, the availability of therapies and resources.

Knowledge Summaries are a resource to help reinforce or inform decision making. They do not override the responsibility or judgement of the practitioner to do what is best for the animal in their care.

## The evidence

All three studies (Heur et al., 2024, King et al., 2018, and Noli et al., 2017) present strong evidence proving that single or two-dose treatments are as efficacious as daily treatment for canine otitis externa. The study designs were all randomised control trials with King et al. (2018) and Heur et al. (2024) being single blinded while Noli et al. (2017) was a randomised control non-blinded trial. Randomised control studies are one of the higher levels of evidence, as they minimise bias and allow for a more reliable comparison. While blinding does aid in lowering bias, it can be difficult or obstructive to have full blinding in these studies due to certain aspects, such as working with owners who must be the ones to administer treatment. Two-dose veterinary administered treatments showed a faster pruritus reduction. However, by the end of the study both forms of treatments were valued to be equally effective (Heur et al., 2024). Across studies, relapse rates were low (around 10–11%) for all treatments. However, there were potential biases from unblinded owners (King et al., 2018), variability in bacterial resistance (such as *Pseudomonas aeruginosa*) (King et al., 2018), and inconsistency in ear cleaning protocols used in conjunction with prescription therapy between Heur et al. (2024), King et al. (2018), and Noli et al. (2017). The consistency of objective outcomes like otitis index scores (OTIS-3) and the large sample sizes in the King et al. (2018) and Heur et al. (2024) studies strengthen the evidence.

## Summary of the evidence

## Table 1

**Heur et al. (2024):** Clinical safety and efficacy of a single-dose gentamicin, posaconazole and mometasone furoate otic suspension for treatment of canine otitis externa **Aim: **To develop a single-dose, in-clinic, veterinary professional-administered treatment for canine otitis externa to improve compliance and canine welfare.

Population:	Dogs diagnosed with acute or recurrent canine otitis externa in one or both ears at 35 veterinary clinics in France, Germany, and the Netherlands.
Sample size:	316 client-owned dogs (276 included).
Intervention details:	Group A (143 dogs): 153 dogs were originally enrolled in Mometamax group, 10 were not included in the study based on the set exclusion criteria. Single application of 0.8 ml of Mometamax^®^ Ultra on day 0 by veterinary staff. Ears were cleaned using saline and allowed to dry prior to application. Group B (133 dogs): 163 dogs were originally enrolled in Osurnia group but 30 were not included in the study based on the set exclusion criteria. Single application of 1 ml of Osurnia^®^ on days 0 and 7 by veterinary staff. Ears were not cleaned prior to treatment.
Study design:	Multicentre, randomised, examiner-masked, controlled trial.
Outcome Studied:	A clinical ear score otitis index scores (OTIS-3) based on ear erythema, oedema/swelling, exudate quantity, and ear lesion/ulceration. Scores determined on days 0, 7, 14, 28, and 42.
Main Findings (relevant to PICO question):	Treatment success was based on an ear score of 4 or greater on day 14 and 3 or greater at day 28. 128/143 dogs (89.5%) receiving a single application of Mometamax^®^ Ultra on day 0 experienced treatment success. 116 of the 133 dogs given a single application of Osurnia^®^ on days 0 and 7 were successfully treated (87.2%). The lower confidence limit of the primary treatment success endpoint (clinical ear score) was found to be -0.0590, indicating that the effectiveness of the Mometamax^®^ treatment is comparable to the effectiveness of the Osurnia^®^ The p-value was found to be less than 0.0001, suggesting the results are statistically significant and the differences between the treatments are likely not due to chance. The relapse rate was determined based on the number of dogs with an ear score of 5 or greater on day 42 after treatment success on day 28. The lower confidence limit for relapse rate on day 42 was found to be -0.0952, which suggests that the Mometamax^®^ treatment may have a lower relapse rate than the Osurnia^®^ These results suggest that the Mometamax^®^ treatment is at least as effective as the Osurnia^®^ treatment in preventing relapse of otitis externa.
Limitations:	There was no subjective outcome being studied. Ear swab samples were taken on days 0 and 28 for microbial culture and antibiotic and antifungal susceptibility testing. The authors did not elaborate on the results of the ear swabs. It would have been beneficial if the authors used the results of the ear swabs to create a subjective outcome to study, such as a microbial count. The impacts of ear cleaning being completed in one group and not in the other were not evaluated.

## Table 2

**King et al. (2018):** A randomized, controlled, single-blinded, multicenter evaluation of the efficacy and safety of a once weekly two dose otic gel containing florfenicol, terbinafine and betamethasone administered for the treatment of canine otitis externa **Aim: **To improve compliance and convenience in the treatment of canine otitis externa with a novel otic gel applied to the ear canal twice at a one-week interval, while maintaining safety and efficacy.

Population:	Dogs diagnosed with acute or recurrent canine otitis externa in one or both ears from 30 first opinion veterinary practices in France, Germany, and the United Kingdom.
Sample size:	285 client-owned dogs.
Intervention details:	Group A (n = 148 dogs): Ears cleaned with physiological saline to remove otic debris and permit visualisation of tympana. Each affected ear would receive formulation of 1 ml of a viscous gel (Osurnia^®^ otic gel) 1% florfenicol, 1% terbinafine and 0.1% betamethasone acetate. Dogs returned in 7 days for retreatment. Clinical rechecks completed on days 28 and 56, animals were evaluated using the OTIS-3 scoring system. Group B (n = 137 dogs): Owners were sent home with instructions to administer the otic suspension once daily for four more days. Each affected ear was treated with 1 ml of an otic suspension containing 1.11 mg/ml hydrocortisone aceponate, 15.1 mg/ml miconazole and 1505 I.U./ml of gentamicin. In this control group, ears were cleaned with physiological saline to remove otic debris and permit visualisation of tympana. Dogs were reevaluated after 7 days. Clinical rechecks completed on days 28 and 56, animals were evaluated using the OTIS-3 scoring system.
Study design:	Randomised, positive-controlled, single-blinded, multicentre trial.
Outcome Studied:	The frequency (percentage) of dogs with either a bacteriological or fungal response at day 28 or day 56. The decrease of bacterial or fungal counts on cytology at day 28 and day 56. Percentage reduction in OTIS-3 at day 56 compared to baseline. Percentage of dogs with an OTIS-3 ≤ 3 (considered a clinical success) at day 28 and day 56. Primary outcome measured was percentage reduction in otitis index scores (OTIS-3) at day 28 compared to day 0. The overall assessments by the owners and investigators at day 28 and day 56. Decrease in pain assessed by the investigator at day 28 and day 56. Decrease in pain and pruritus assessed by the owner at day 28 and day 56. Speed of response assessed by reduction in OTIS-3 at day 7.
Main Findings (relevant to PICO question):	Owners reported a significant difference in pruritus (itching) at day 7, in favour of the otic suspension treatment. The mean OTIS-3 stayed low until day 56 in both groups. The OTIS-3 decreased on average by 62.5% and 63.6% for the otic gel and by 63.4% and 60.5% for the otic suspension on day 28 and day 56. Three quarters of clinicians and owners reported a good to excellent response of otitis externa to the otic gel administration after day 28, which was not significantly different to the suspension. Speed of response (in terms of absolute and percentage reduction in OTIS-3 and owner pruritus VAS) for the daily treatment was significantly better than the Osurnia^®^ otic gel at day 7. P-values at baseline were found to be above 0.05 which shows no difference between the animals. P-values comparing Osurnia^®^ otic gel versus otic suspension all except day 7 were non-significant. At day 7 there was a favourable outcome for otic suspension. No significant differences in pain or pruritus scores were observed between the two groups on day 28 or day 56. On day 56, there were no differences between the group treated with the gel and that with the suspension in owners and investigators overall assessment scores. Relapses were recorded at day 56 in 11 dogs treated with the Osurnia^®^ otic gel, and in 10 dogs treated with otic suspension. The cytology counts for bacteria, fungi and neutrophils decreased in both groups, with no significant difference seen between groups. The study revealed non-inferiority therefore, the CI range passed through zero which indicated there may not be a significant effect between the two treatments. The confidence interval found was -6.1–7.9%. No effect size given.
Limitations:	The study design did not allow the possibility of blinding owners due to the nature of treatment. This could potentially lead to bias in reporting of treatment. Different treatment responses caused by resistant bacteria *Pseudomonas aeruginosa*. Authors are employees of Elanco animal health and funding was provided by Novartis which is the owner of Elanco.

## Table 3

**Noli et al. (2017):** Impact of a terbinafine-florfenicol-betamethasone acetate otic gel on the quality of life of dogs with acute otitis externa and their owners **Aim: **To evaluate otic treatment administered by veterinarians on the quality of life of dogs with otitis externa and their owners, compared to daily at home owner-administered treatment.

Population:	Dogs diagnosed with acute or recurrent canine otitis externa in one or both ears in multiple Italian veterinary centres.
Sample size:	50 client-owned dogs.
Intervention details:	Group A (n = 25 dogs): Single application of 1 ml of Osurnia^®^ otic gel on days 0 and 7 by a veterinarian. Ear was cleaned before application by clinician but not again until end of study. No treatment at home by owner. Group B (n = 25 dogs): Once daily application of Posatex^®^ by owner for 14 days. Ear cleaned by clinical before first application. Biweekly ear cleaning by owner at home using Surosolve™ during a 14-day period.
Study design:	Multi-centre, randomised, non-blinded, controlled trial.
Outcome Studied:	Otitis using the validated, objective clinical scoring system (OTI-3 scale) and semiquantitative cytological examination on days 0, 7, 14 and 28. Quality of life of owner and dog using a validated questionnaire on days 0, 7, 14, and 28. Pruritus using a Visual Analog Scale (VAS) on days 0, 7, 14, and 28.
Main Findings (relevant to PICO question):	Higher proportional improvement of VAS pruritus on day 7 in Group A compared to Group B, but not on days 14 and 28; both treatments were equally effective at decreasing pruritus but the Osurnia^®^ otic gel had a more rapid onset of action. No difference in OTI-3 percentage improvement between Group A and Group B. Cytology scores on days 7 and 14 were greater in Group A compared to Group B with the exception of day 28.
Limitations:	Recurrence was not evaluated in this study. All dogs were sent home with owners during the duration of the study. This creates a potential risk for the dogs to be exposed to external factors that might skew the results of the trial. The study does not explicitly calculate or report effect size, it only focuses on statistical significance through p-values to assess the different between the treatment groups. Study states that ‘statistical signiﬁcance was set as a two-sided P-value < 0.05;’ however, the specific study values are not provided. The study does not report confidence intervals for the results.

## Appraisal, application and reflection

Otitis externa treatments combine ear cleaning with a topical medication that contains a combination of an antibiotic, an antifungal, and a corticosteroid. Two of the articles, Heur et al. (2024) and King et al. (2018) used physiologic saline to flush and clean ears affected with otitis externa in canine patients within the study. This cleaner worked to mechanically remove debris from the external ear canal but did not have antimicrobial activity. In the Noli et al. (2017) study, Surosolve™ (a product that contains the antimicrobials salicylic acid and chloroxylenol) or Otoact^®^ (a cleaning product that contains squalene, a cerumenolytic) were utilised. These products cleaned the ears by mechanical means but also had some unmeasured antimicrobial effect. All three articles examined the efficacy of Osurnia^®^ otic gel, a topical aural medication that contains the broad spectrum antibiotic ﬂorfenicol, antifungal terbinaﬁne, and steroid betamethasone acetate. Noli et al. (2017) studied the impact of Osurnia^®^ otic gel on quality of life. Heur et al. (2024) compared Osurnia^®^ to Mometamax^®^ Plus, an otic suspension containing the broad spectrum topical antibiotic gentamicin, antifungal posaconazole, and steroid mometasone furoate. King et al. (2018) compared the efficacy of two-dose Osurnia^®^ otic gel with multidose Easotic^®^, an otic suspension containing the broad-spectrum antibiotic gentamicin sulfate, antifungal miconazole nitrate, and steroid hydrocortisone aceponate.

Overall, these studies contribute important findings regarding the efficacy of single or two-dose and daily multi-dose prescription treatments for otitis externa in dogs. Heur et al. (2024) demonstrated that Mometamax^®^ (single-dose) was successful in resolving otitis externa in 128 out of 143 dogs, while Osurnia^®^ (two-dose) showed success in 116 out of 133 dogs, with no difference in relapse rates between the treatment protocols. King et al. (2018) provided additional insights, noting a slight improvement in the secondary endpoint of speed of response at day 7 with daily treatment; however, there were no significant differences in cytology counts for bacteria, fungi, and neutrophils, nor in pain or pruritus scores between treatment groups at day 28 or 56, suggesting equal efficacy overall. The researchers recorded relapses at day 56 in 11% of dogs treated in accordance to the once weekly two dose otic gel protocol and also in 11% of the dogs treated in accordance to the daily otic suspension treatment protocol. There was no significant difference between the Groups; however, the study did not explore long-term follow-up or stratified risk factors. Lastly, Noli et al. (2017) showed that while both treatments were effective at reducing pruritus, Group A (Osurnia^®^ otic gel) provided faster improvement in Visual Analog Scale (VAS) score with improved pruritus relief by day 7 as compared to Group B (Posatex^®^). By days 14 and 28, both treatments were equally effective, showing no long-term advantage for either. Therefore, both treatments were equally effective at decreasing pruritus with the only exception of the Osurnia® otic gel having a more rapid onset of action. In addition, there was no difference in OTI-3 percentage improvement between Group A and Group B. The cytology scores on days 7 and 14 were greater in Group A compared to Group B with the exception of day 28. Collectively, these studies suggest that while some treatments may offer quicker relief, they all demonstrate similar long-term efficacy. Of additional note, in the Noli et al. (2017) study, there was no separation of data for dogs experiencing first-time otitis externa occurrence compared to dogs experiencing a recurrence.

Single or two-dose veterinary-administered versus daily prescription multidose treatment regimens for otitis externa are both widely available in general veterinary practice. Daily, multidose products are applied directly into affected ear canals, generally by an animal’s owner or caretaker once or twice per day over five to seven or more days. Daily treatment protocols can be uncomfortable for the dog and difficult or even sometimes dangerous for a client depending on an animal’s temperament and tolerance (Heur et al., 2024). Daily multidose protocols generally consist of a combination of ear cleaning to remove debris and the application of a prescription product. This product often contains a combination of antibiotic, antifungal, and steroid medications. Dosing of daily multidose protocols is usually through drop application based on the weight of an animal. At home treatment compliance is paramount to the success of multidose treatment of otitis externa (Noli et al., 2017). In two-dose protocols, ear cleaning followed by administration of the therapeutic agent occurs in the clinic setting. In this way, single or two-dose therapies can improve safety and compliance barriers to the effective treatment of otitis externa in dogs (Heur et al., 2024). Further, single and two-dose treatments are generally formulated into single use standardised volume ampules rather than relying on the drop dosing of multi dose administrations,resulting in improved standardisation of care and consistent appropriate medicine application and a decreased risk of reinfection or cross contamination (King et al., 2018). Appropriate dosing of antimicrobials is necessary to achieve the minimum inhibitory concentration for aural bacterial and fungal pathogens (Heur et al., 2024).

The key takeaway from the appraisal is that the studies confirm both treatment methods for otitis externa in dogs are effective, with high success rates and similar relapse rates, making both reliable treatment options. Single or two-dose veterinary-administered therapy may offer improved compliance, improve the ease of application, and provide faster initial alleviation of clinical signs including pruritus, which can be beneficial for both the dog and the owner. The published evidence suggests there is no discernable difference in treatment success between the two approaches. Ultimately, single and two-dose veterinary-administered treatment therapies offer comparable long-term effectiveness to multidose daily prescription treatments with no significant evidence of difference in relapse rates reported, adding to their value in managing otitis externa in a safe and effective manner for continuous relief. Therefore, practising veterinarians should choose to prescribe the treatment that best suits the needs of both the client and the patient.

## Methodology

**Search Strategy:** 

Databases searched and dates covered:	CAB Abstracts on CABI Digital Library: [January 2008–February 2025] Pubmed Database via the NCBI website: [2012–2025]
Search strategy:	CAB Abstracts on CABI Digital Library: Title:(canine otitis externa) AND AllField:(Florfenicol, terbinafine, mometasome, furoate) OR AllField:(Gentamicin Sulfate, Betamethasone Valerate, and Clotrimazole, Ointment) OR AllField:(ear infection) AND AllField:(treatment) AND AllField:(topical) AND Title:(osurnia) OR Title:(Mometamax) OR Title:(terbinafine, florfenicol, betamethasone acetate) AND AllField:(dogs) PubMed: ((Canine) OR (Dog)) AND ((Otitis Externa) OR (Ear infection)) AND ((multi dose) OR (multi dose) OR (single dose)) AND (otic)
Dates searches performed:	25 February 2025

**Exclusion / Inclusion Criteria:** 

Exclusion:	Studies relating to species other than dogs; not analysing otitis externa; uses commercial over-the-counter treatments; not relevant to PICO question; do not include medications on our search string.
Inclusion:	Comparison of single or two-dose vs multi dose treatments with regards to otitis externa.

**Search Outcome:** 

Database	Number of results	Excluded – not looking at result of multi or single doses	Excluded – studies relating to species other than dogs	Total relevant papers
CAB Abstracts	5	1	3	1
PubMed	3	1	0	2
Total relevant papers when duplicates removed				3

## Acknowledgements

Thank you to Dr. Rachael Kreisler for her contributions and oversight to the student doctors through their Principals of Veterinary Scholarship course.

## Author contributions

**Ashlee Minne** acted as Methodology (lead) and collaborated equally with **Isabella Alarcon**, **Garen Burch**, **Stephanie Kay**, **Osdel Macides**, and **Caroline Thompson** in Conceptualisation and Writing – Original draft preparation. **Dr. Mariah Zeigler** is the Corresponding Author and provided Faculty Supervision and served as Primary Investigator. **Dr. Zeigler** oversaw Data curation, Writing – Original draft preparation and methodology (supporting). Additionally, **Dr. Zeigler** led Revisional Writing – Review & Editing (lead).

## ORCiD

Ashlee Minne: https://orcid.org/0009-0002-3022-7954

Isabella Alarcon: https://orcid.org/0009-0003-1426-1172

Garen Burch: https://orcid.org/0009-0008-0703-5253

Stephanie Kay: https://orcid.org/0009-0005-6471-5544

Osdel Macides: https://orcid.org/0009-0008-9882-2024

Caroline Thompson: https://orcid.org/0009-0006-8966-1375

Mariah K. Zeigler: https://orcid.org/0000-0002-1045-1791

## Conflict of Interest

The authors declare no conflicts of interest.

## Contribute to the evidence

There are two main ways you can contribute to the evidence base while you are enhancing your CPD:

- Tell us your information need
- Write a Knowledge Summary

Either way, you will be helping to add to the evidence base, and strengthen the decisions that veterinary professionals around the world make to give animals the best possible care. Learn more here: https://veterinaryevidence.org/index.php/ve/author-hub
